# Platelet-to-Lymphocyte Ratio as an Independent Factor Associated With Atrial Tachyarrhythmia

**DOI:** 10.7759/cureus.46775

**Published:** 2023-10-10

**Authors:** Cheng Chen, Xinyan Tang, Ping Fan

**Affiliations:** 1 Department of Medical Sciences, Yangzhou Polytechnic College, Yangzhou, CHN; 2 Department of Family Medicine, Jiangsu Vocational College of Medicine, Yancheng, CHN; 3 Department of Cardiology, The Air Force Hospital From Eastern Theater of the People's Liberation Army, Nanjing, CHN

**Keywords:** platelet-to-lymphocyte ratio, receiver operating characteristic curve, risk factor, inflammation, atrial tachyarrhythmia

## Abstract

Objective

To investigate the relationship between the presence of atrial tachyarrhythmia (AT) and the platelet-to-lymphocyte ratio (PLR), which is a recently described inflammatory marker.

Methods

A total of 149 patients with AT and 187 healthy volunteers were included in this study. Complete blood count, serum lipids, and serum creatinine were tested, and dynamic electrocardiograms were performed routinely in all subjects. Student’s t-test, Mann-Whitney U test, logistic regression analysis, and receiver operating characteristic curve analysis were used for statistical analysis.

Results

In the AT group, the proportions of patients with diabetes, hypertension, and coronary heart disease were higher than those in the control group. Higher blood platelet, low‐density lipoprotein, neutrophil-to-lymphocyte ratio, and PLR were detected in the AT group. In addition, haemoglobin, lymphocytes, and the fastest ventricular rate were significantly lower in the AT group. Higher PLR was identified as independently associated with the presence of AT. When a cut-off value of 119.47 was used, the sensitivity and specificity of PLR for predicting AT were 79.2% and 81.3%, respectively.

Conclusion

Elevated PLR was associated with AT, suggesting that it might be useful in the future as an adjunct biomarker for the detection of the disease.

## Introduction

Atrial tachyarrhythmia (AT) is an atrial arrhythmia with an atrial rate greater than 100 beats/minute. AT, characterized by abnormal, disorganized, and rapid atrial electrical activity, is a major public health problem [1]. The estimated incidence of AT in the general population is between 0.34% and 0.46% [2]. Atrial tachycardia can affect the quality of life of patients and lead to abnormal left ventricular function and cardiomyopathy. In addition, AT induces embolic events and stroke.

An increase in the count of blood cells and subtypes of leukocytes is an important cardiovascular risk factor [3,4]. Previous studies have shown that inflammation acts as a facilitator in the induction of supraventricular tachycardia (SVT), and increased inflammatory markers may have a role in predicting AT in patients without coronary artery disease [5]. Aydın et al. examined the neutrophil-to-lymphocyte ratio (NLR) as a potential predictor of SVT [6]. In addition, the platelet-to-lymphocyte ratio (PLR) is related to atrial thrombus and atrial fibrillation [7,8]. AT is common and associated with bad prognosis [9], and there are no studies on the relationship between PLR and AT. Therefore, in this study, we aimed to investigate the relationship between PLR and AT.

## Materials and methods

The study design was a retrospective cross-sectional study. It was conducted in the Department of Cardiology of the Air Force Hospital from Eastern Theater of the People's Liberation Army between January 2010 and December 2020. The study participants consisted of 149 paroxysmal focal AT patients over 18 years of age, while age- and sex-matched healthy control volunteers (n = 187) were recruited to act as controls. The Helsinki Declaration principles were followed in the investigation. The study was approved by the ethics committee of the Air Force Hospital from Eastern Theater of the People's Liberation Army (0406001), and each participant signed a written informed consent before taking part in the study.

The exclusion criteria were as follows: the left atrium had a diastolic diameter over 50 mm; the patient took antiarrhythmics in the past six months; the patient has been diagnosed with paroxysmal or permanent atrial fibrillation and atrial flutter; patients with the presence of permanent pacemaker, acute coronary syndrome, congenital cardiac disease, New York Heart Association (NYHA) class IV heart failure, active inflammatory and infectious diseases, severe kidney failure, severe abnormal liver function, thyroid disorder, malignancies, and alcohol consumption; and patients who were pregnant.

Age, sex, history of coronary heart disease, hypertension, and diabetes were recorded for each patient. Serum low‐density lipoprotein (LDL), serum high‐density lipoprotein (HDL), serum creatinine (Cr), blood leukocytes, haemoglobin, blood platelets, and blood neutrophils were reviewed. With the help of ECGLAB dynamic electrocardiogram analysis software (Beijing MEIGAOYI Software Technology Co., Ltd., Beijing, China), the dynamic electrocardiogram characteristics were recorded. Analyses of the dynamic electrocardiograms were performed for all patients and the control population to determine the occurrence of AT, fastest ventricular rate, and slowest ventricular rate.

The data were exported to SPSS version 22.0 software (IBM Corp., Armonk, NY) for analysis. The data were analysed as follows: (1) chi-square (χ2) analysis was used to determine the relationships between categorical variables. (2) Mann-Whitney U tests were used to compare non-normally distributed variables, and independent samples t-tests were used if the dependent variables were continuous and approximated a normal distribution. (3) Univariate and multivariable logistic regression analyses were performed to identify risk factors associated with AT. A P-value of <0.05 was considered significant. (4) Receiver operating characteristic (ROC) curve analysis was used to determine the suitable PLR cut-off value for predicting AT.

## Results

The study population consisted of 336 subjects (149 subjects in the AT group and 187 subjects in the control group). The baseline demographic, biochemical, and dynamic electrocardiogram characteristics of the study population are shown in Table 1. There were no statistically significant differences between the groups with respect to age, sex, HDL, Cr, white blood cells (WBC), neutrophil, or slowest ventricular rate. The proportions of patients with diabetes, hypertension, and coronary heart disease were significantly higher in the AT group than in the control group. The AT group had lower haemoglobin (P = 0.006), lower lymphocytes (P = 0.000), and a lower fastest ventricular rate (P = 0.006). The AT group showed a higher blood platelet count (P = 0.000), LDL level (P = 0.001), NLR ratio (P = 0.001), and PLR ratio (P = 0.001) (Table 1).

**Table 1:** Baseline characteristics of the control and AT groups Data are shown as mean ± standard deviation, median (interquartile range), and frequency (percentages). DM, diabetes mellitus; PLR, platelet-to-lymphocyte ratio; NLR, neutrophil-to-lymphocyte ratio; LDL, low-density lipoprotein; HDL, high-density lipoprotein; AT, atrial tachyarrhythmia.

	Control group	AT group	t/X^2^/Z	P
Age (year）	70.76±13.57	73.66±13.85	-1.927	0.055
Male (%)	89 (46.35）	65 (45.13）	0.049	0.825
DM (n, %)	93 (48.32）	87 (60.41）	4.747	0.029
Hypertension (n, %)	102 (53.12）	92 (63.88）	3.907	0.048
Coronary heart disease (n, %)	88 (45.83）	86 (58.90）	5.673	0.017
LDL (mmol/L)	2.19±0.66	2.33±0.84	-3.425	0.001
HDL (mmol/L)	1.32±0.36	1.33±0.45	-0.01	0.992
Creatinine (μmol/L)	78.3 (29）	80.2 (32.9）	-0.414	0.679
Haemoglobin (g/L)	122.26±16.08	117.42±15.73	2.766	0.006
White blood cells (× 10^9^/L)	6.31±0.66	6.29±2.78	0.081	0.935
Platelets (× 10^9^/L)	186.21±60.55	222.33±71.36	-5.061	0.000
Neutrophil (× 10^9^/L)	3.35±1.11	3.29±1.23	0.403	0.687
Lymphocyte (× 10^9^/L)	2.03±0.56	1.37±0.48	11.336	0.000
NLR	1.78±0.80	2.86±2.25	-5.575	0.000
PLR	95.68±34.72	181.92±94.82	-10.548	0.000
Fastest ventricular rate (beats per minute)	107.13±20.19	101.30±18.27	2.742	0.006
Slowest ventricular rate (beats per minute)	50.37±11.53	49.02±11.19	1.083	0.280

As a result of the univariate logistic regression analysis, higher platelets (OR = 1.009, 95% CI: 1.005-1.013), higher NLR (OR = 2.172, 95% CI: 1.678-2.811), higher PLR (OR = 1.038, 95% CI: 1.029-1.048), higher proportion of diabetes (OR = 1.644, 95% CI: 1.063-2.543), hypertension (OR = 1.645, 95% CI: 1.058-2.558), and history of coronary heart disease (OR = 1.613, 95% CI: 1.045-2.490) were related to AT. In addition, lower lymphocyte levels (OR = 0.085, 95% CI: 0.047-0.152), lower haemoglobin levels (OR = 0.981, 95% CI: 0.967-0.995), and lower fastest ventricular rates (OR = 0.984, 95% CI: 0.972-0.996) were associated with AT (Table 2).

**Table 2:** Univariate logistic regression for the association between risk factors and AT PLR, platelet-to-lymphocyte ratio; NLR, neutrophil-to-lymphocyte ratio; LDH, lactate dehydrogenase; AT, atrial tachyarrhythmia.

	OR	95% CI	P
LDH	1.274	0.956-1.698	0.098
Haemoglobin	0.981	0.967-0.995	0.007
Hypertension	1.645	1.058-2.558	0.027
Diabetes	1.644	1.063-2.543	0.025
Platelets	1.009	1.005-1.013	0.000
Lymphocyte	0.085	0.047-0.152	0.000
NLR	2.172	1.678-2.811	0.000
PLR	1.038	1.029-1.048	0.000
Coronary heart disease	1.613	1.045-2.490	0.031
Fastest ventricular rate	0.984	0.972-0.996	0.008

Factors such as history of coronary heart disease, diabetes, hypertension, lactate dehydrogenase (LDH), haemoglobin, blood platelets, lymphocytes, NLR, PLR, and fastest ventricular rate were included in the multivariable logistic regression analysis. In the multivariate logistic regression analysis, PLR (OR = 1.032, 95% CI: 1.004-1.062) was an independent risk factor for AT (Table 3).

**Table 3:** Multivariate logistic regression for the association between risk factors and AT PLR, platelet-to-lymphocyte ratio; NLR, neutrophil-to-lymphocyte ratio; AT, atrial tachyarrhythmia.

	OR	95% CI	P
Haemoglobin	0.987	0.967-1.007	0.202
Hypertension	1.804	0.964-3.374	0.065
Diabetes	0.556	0.298-1.037	0.065
Platelets	1.000	0.984-1.017	0.965
Lymphocyte	0.382	0.053-2.725	0.337
NLR	1.024	0.692-1.515	0.905
PLR	1.032	1.004-1.062	0.027
Coronary heart disease	1.040	0.543-1.994	0.905
Fastest ventricular rate	1.003	0.982-1.024	0.801

**Figure 1:**
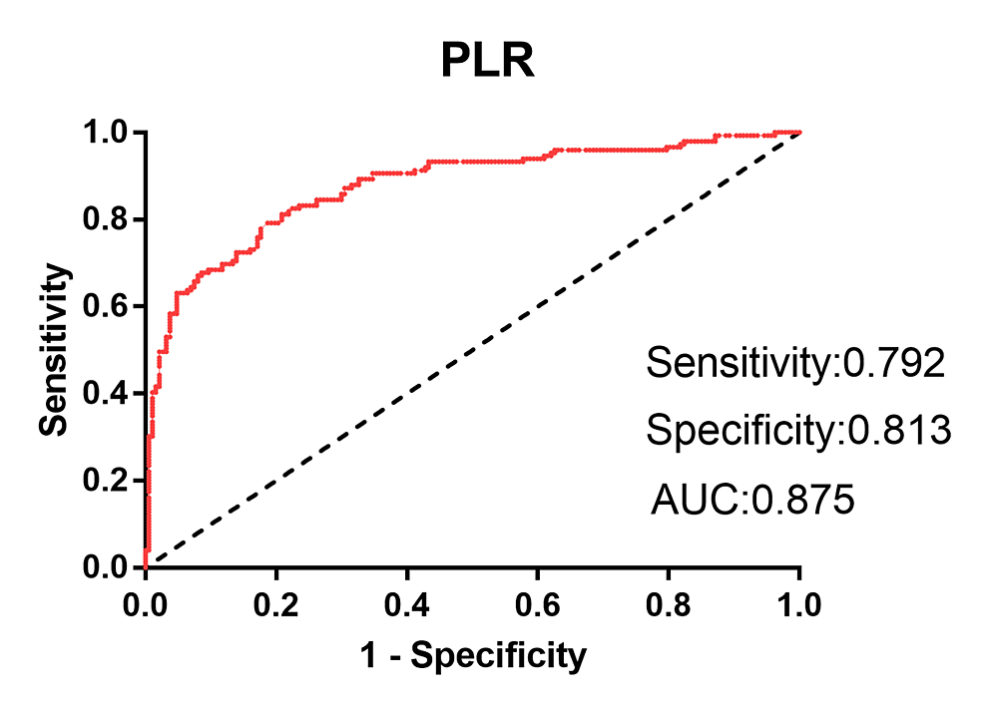
Receiver operating characteristic curve analysis of the platelet-to-lymphocyte ratio data for the presence of AT AUC, area under the curve; AT, atrial tachyarrhythmia; PLR, platelet-to-lymphocyte ratio.

The ROC curve analysis indicated that the optimal PLR cut-off value for predicting AT was 119.47, with a 79.2% sensitivity and an 81.3% specificity (area under the curve (AUC) = 0.875, 95% CI: 0.837-0.914) (Figure 1).

## Discussion

AT is a common arrhythmia that often occurs in patients with structural heart disease with or without heart failure and ischaemic coronary disease. The aetiology of AT is related to hypoxia, lung disease, ischaemic heart disease, heightened sympathetic tone, metabolic disorders, and intake of stimulant foods such as cocaine, caffeine, alcohol, metabolic disorders, nicotine, and ephedra [10].

In our study, we found that the proportions of patients with DM, hypertension, and a history of coronary heart disease were significantly higher in the AT group than in the control group. In addition, LDH, platelets, and NLR in the AT group of patients were significantly higher than those in the control group. We also found that haemoglobin, lymphocytes, and the fastest ventricular rate in the AT group of patients were significantly lower than those in the control group.

AT is very common in patients with heart failure, and heart failure patients are often accompanied by cardiac structural abnormalities [11]. Moreover, AT is closely related to inflammation [12]. Inflammation can lead to inflammatory factor secretion disorders and affect the function of cardiomyocytes through various pathways. Inflammatory factors cause calcium overload and a prolonged action potential duration [13]. In addition, inflammatory factors reduce the ATPase activity of the sarcoplasmic reticulum in cardiomyocytes, resulting in calcium ion efflux from the sarcoplasmic reticulum, which further increases the intracellular calcium load in cardiomyocytes [14]. Inflammatory factors also induce sodium channel abnormalities, resulting in abnormal depolarization of cardiomyocytes [15]. Furthermore, cardiomyocytes can release inflammatory factors such as interleukin-18 (IL-18) and tumour necrosis factor-α (TNF-α) and affect cardiac function [16,17]. Studies have confirmed that activation of the coagulation cascade occurs during the systemic inflammatory response and induces AT through a series of direct or indirect pathways [18]. Moreover, the systemic inflammatory response leads to changes in the haemodynamics and blood flow distribution in various organs and tissues, which induces cardiac ischaemia, hypoxia, and the production of proarrhythmic factors. This may eventually lead to enhanced automaticity, reentry, and triggered activity [12]. In a systemic inflammatory state, the levels of inflammatory factors, such as TNF-α, interleukin-6 (IL-6), and interleukin-8 (IL-8), that reflect acute infection are significantly increased. Some scholars considered that the level of inflammatory factors reflecting acute infection may also have predictive value for AT [19].

The PLR ratio is a novel biomarker of systemic inflammation and is calculated by dividing the number of platelets by the number of lymphocytes [20]. Lymphocyte count is an early marker of systemic inflammation [21]. Decreased lymphocyte counts often indicate physiological stress and worsening health. Changes in the number of lymphocytes provide more detailed information about the health status of the organism compared to leukocytes [22]. Increased platelet counts have been associated with an elevated risk of thrombotic diseases. Increased platelet counts in the circulation lead to the formation of atherosclerotic plaques [23]. Platelets can also release a variety of proinflammatory cellular and chemical factors [24].

Elevated platelet levels and decreased lymphocyte levels are risk factors for malignant cardiovascular events [25,26]. PLR is a ratio that is more stable than the single lymphocyte or platelet counts. It may be a better predictor of cardiovascular disease [7]. To our knowledge, few studies have assessed the relationship between PLR and AT. In the present study, PLR, a reliable marker of inflammation, was significantly higher in the AT group than in the control group. Dereli et al. reported that PLR has adequate predictive value for atrial fibrillation recurrence. After electrical cardioversion, 287 atrial fibrillation patients were followed up for six months, and 108 patients experienced atrial fibrillation recurrence [7]. The PLR in the patients with atrial fibrillation recurrence was significantly higher than that in the patients without atrial fibrillation recurrence. The multivariate logistic regression analysis showed that PLR is an independent risk factor for atrial fibrillation recurrence. In addition, PLR has good specificity and sensitivity for predicting the recurrence of atrial fibrillation [7]. In a study carried out by Belen et al., 351 patients with rheumatic mitral stenosis were divided into two groups based on whether they had left atrial thrombus [8]. The level of PLR in patients with left atrial thrombus was significantly higher than that in patients without left atrial thrombus, and PLR was an independent risk factor for left atrial thrombus. Furthermore, PLR has a high value for predicting left atrial thrombus. In our study, the multivariate logistic regression analysis indicated that a higher PLR was an independent risk factor for AT. In addition, we found that the PLR showed a 79.2% sensitivity, an 81.3% specificity, and an AUC of 0.875 (95% CI, 0.837-0.914) to confirm AT, suggesting that the PLR has predictive value for AT.

Many studies have reported that NLR is associated with cardiovascular diseases, such as acute coronary syndromes, cardiac arrhythmias, and atherosclerosis [27,28]. In the present study, the NLR in AT group patients was significantly higher than that in the control group; however, the multivariate logistic regression analysis showed that NLR was not an independent risk factor for AT. Therefore, PLR may act as a biomarker that is closely related to AT. In addition, PLR is a practical and widely utilized parameter requiring no additional costs.

In this study, although PLR shows a good predictive value for AT (cut-off value = 119.47, sensitivity = 79.2%, specificity = 81.3%), a number of limitations are present. First, our study was single-centred, and the number of patients was relatively small. Second, the present study was retrospective in design and had a short duration of follow‐up. Third, we did not detect inflammatory factors such as TNF-α, IL-6, IL-8, and C-reactive protein, and the relationship between PLR and biomarkers of inflammation has not been studied. Furthermore, electrophysiological examinations were not performed on patients. Although this study has limitations, it represents one of the first evaluations of the link between PLR and AT. We confirmed that PLR is a cheap and convenient biomarker for AT.

## Conclusions

In our study, PLR was found to be an independent parameter for the presence of AT. PLR is a readily available and inexpensive parameter that can be used to predict AT. It is necessary to conduct prospective studies in different clinical settings to determine the role of this parameter in AT and other cardiovascular diseases.
